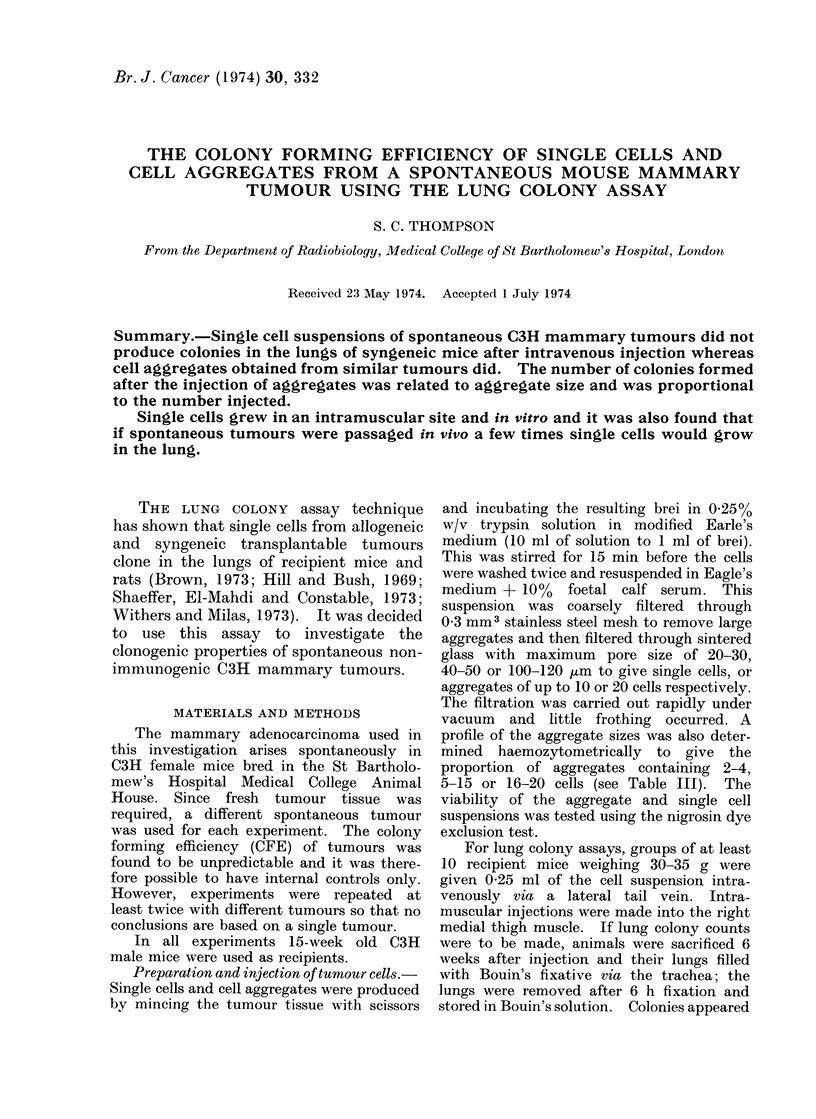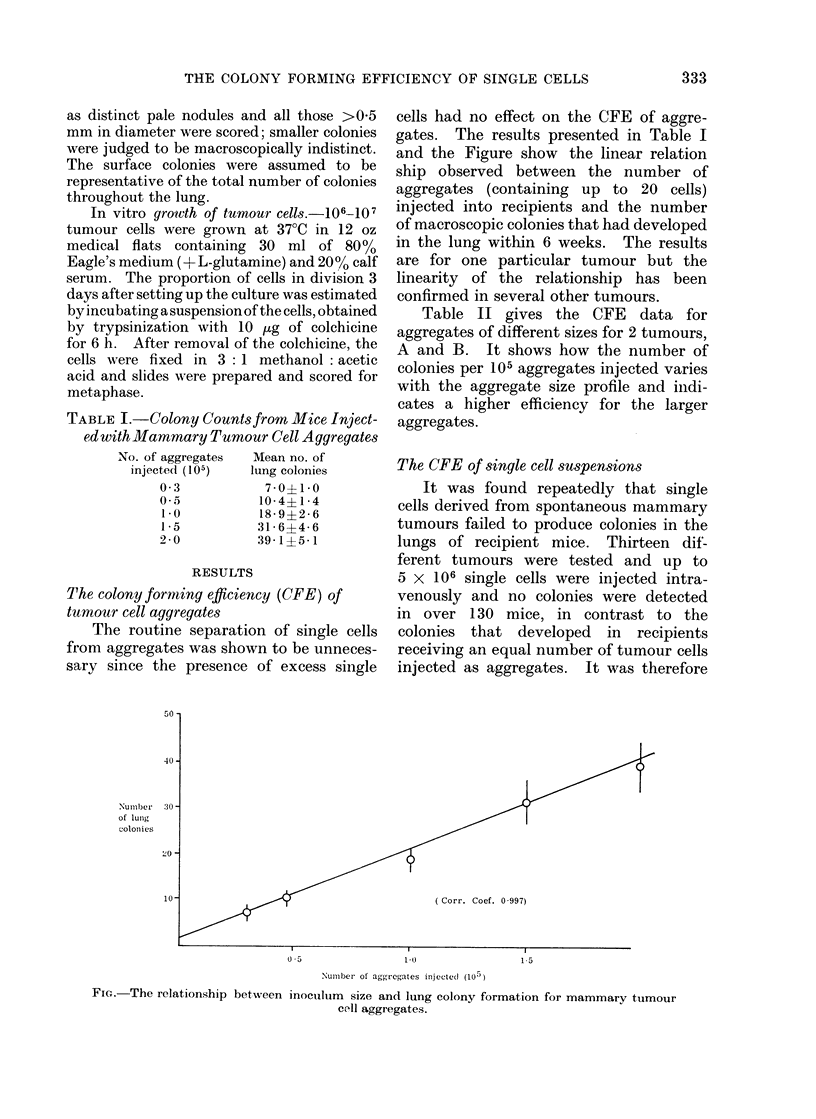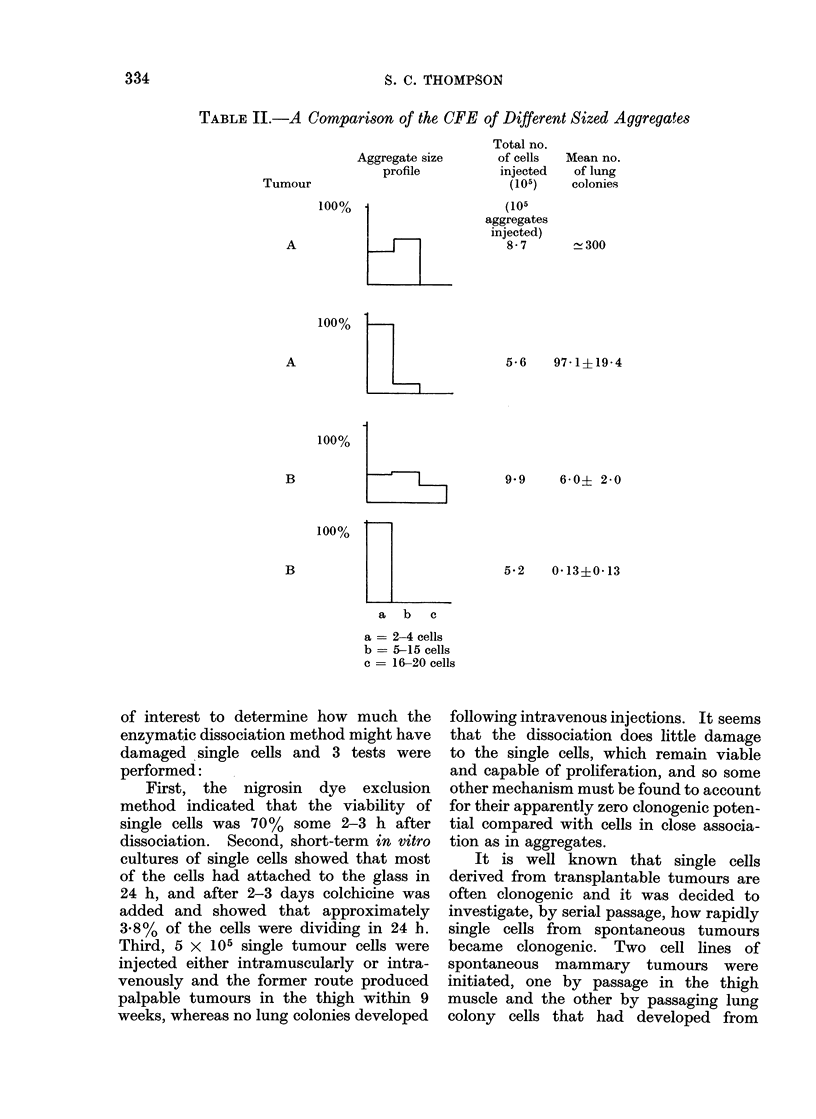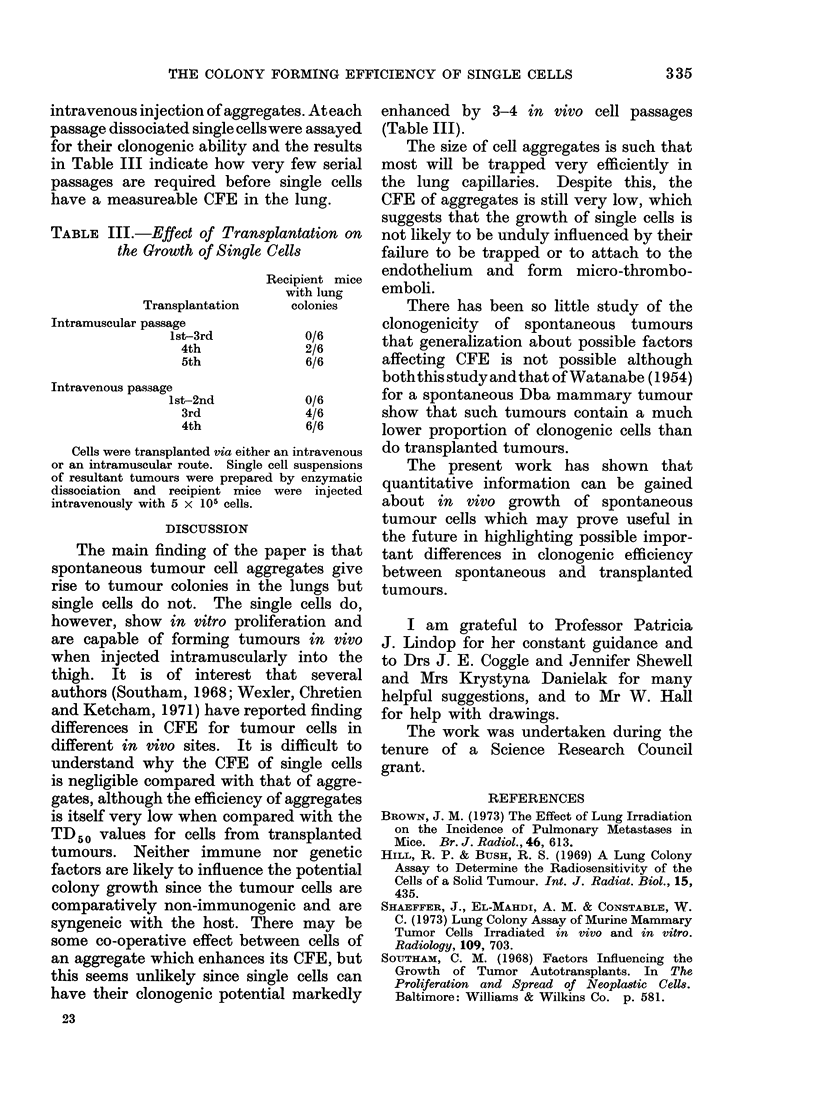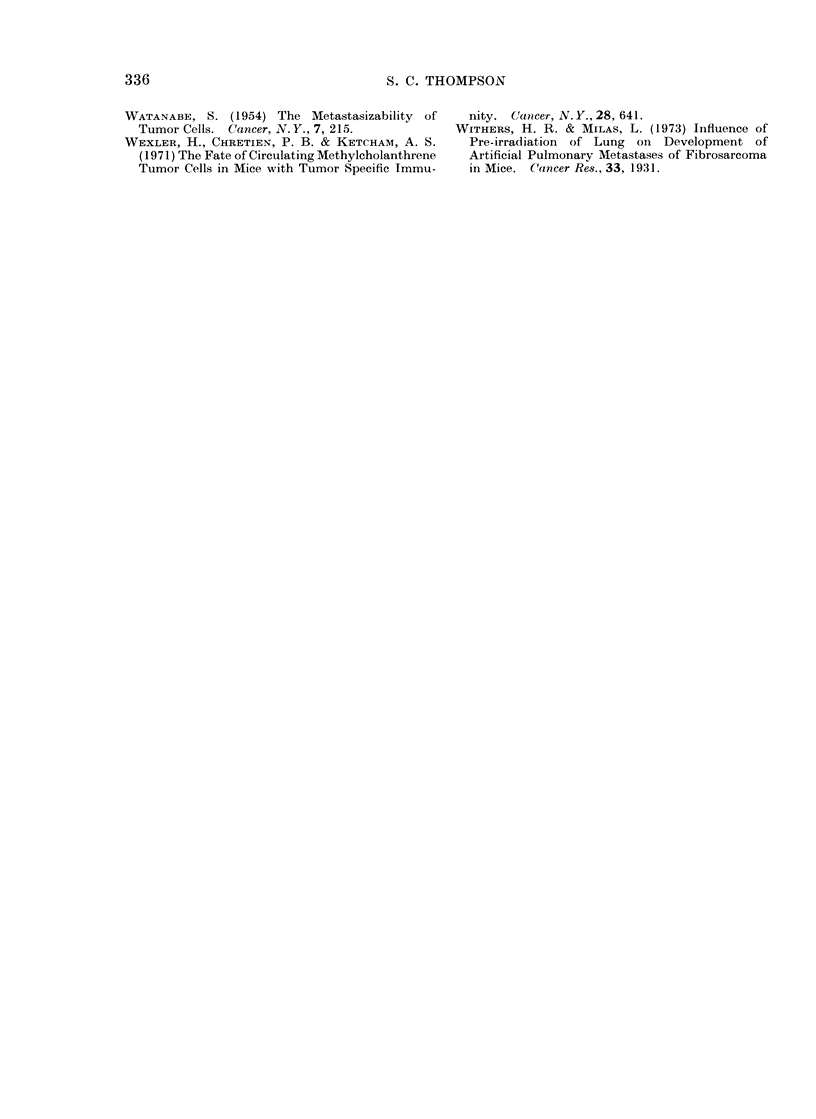# The Colony Forming Efficiency of Single Cells and Cell Aggregates from a Spontaneous Mouse Mammary Tumour Using the Lung Colony Assay

**DOI:** 10.1038/bjc.1974.201

**Published:** 1974-10

**Authors:** S. C. Thompson

## Abstract

Single cell suspensions of spontaneous C3H mammary tumours did not produce colonies in the lungs of syngeneic mice after intravenous injection whereas cell aggregates obtained from similar tumours did. The number of colonies formed after the injection of aggregates was related to aggregate size and was proportional to the number injected.

Single cells grew in an intramuscular site and *in vitro* and it was also found that if spontaneous tumours were passaged *in vivo* a few times single cells would grow in the lung.


*Br. J. Cancer* (1974) **30**, 332

### THE COLONY FORMING EFFICIENCY OF SINGLE CELLS AND CELL AGGREGATES FROM A SPONTANEOUS MOUSE MAMMARY TUMOUR USING THE LUNG COLONY ASSAY

S. C. THOMPSON

*From the Department of Radiobiology, Medical College of St Bartholomew's Hospital, London*

Received 23 May 1974. Accepted 1 July 1974

**Summary.—Single cell suspensions of spontaneous C3H mammary tumours did not produce colonies in the lungs of syngeneic mice after intravenous injection whereas cell aggregates obtained from similar tumours did. The number of colonies formed after the injection of aggregates was related to aggregate size and was proportional to the number injected.**

**Single cells grew in an intramuscular site and *in vitro* and it was also found that if spontaneous tumours were passaged *in vivo* a few times single cells would grow in the lung.**

THE LUNG COLONY assay technique has shown that single cells from allogeneic and syngeneic transplantable tumours clone in the lungs of recipient mice and rats (Brown, 1973; Hill and Bush, 1969; Shaeffer, El-Mahdi and Constable, 1973; Withers and Milas, 1973). It was decided to use this assay to investigate the clonogenic properties of spontaneous non-immunogenic C3H mammary tumours.

### MATERIALS AND METHODS

The mammary adenocarcinoma used in this investigation arises spontaneously in C3H female mice bred in the St Bartholomew's Hospital Medical College Animal House. Since fresh tumour tissue was required, a different spontaneous tumour was used for each experiment. The colony forming efficiency (CFE) of tumours was found to be unpredictable and it was therefore possible to have internal controls only. However, experiments were repeated at least twice with different tumours so that no conclusions are based on a single tumour.

In all experiments 15-week old C3H male mice were used as recipients.

*Preparation and injection of tumour cells.*—Single cells and cell aggregates were produced by mincing the tumour tissue with scissors and incubating the resulting brei in 0·25% w/v trypsin solution in modified Earle's medium (10 ml of solution to 1 ml of brei). This was stirred for 15 min before the cells were washed twice and resuspended in Eagle's medium + 10% foetal calf serum. This suspension was coarsely filtered through 0·3 $mm^3$ stainless steel mesh to remove large aggregates and then filtered through sintered glass with maximum pore size of 20–30, 40–50 or 100–120 $\mu$m to give single cells, or aggregates of up to 10 or 20 cells respectively. The filtration was carried out rapidly under vacuum and little frothing occurred. A profile of the aggregate sizes was also determined haemozytometrically to give the proportion of aggregates containing 2–4, 5–15 or 16–20 cells (see Table III). The viability of the aggregate and single cell suspensions was tested using the nigrosin dye exclusion test.

For lung colony assays, groups of at least 10 recipient mice weighing 30–35 g were given 0·25 ml of the cell suspension intravenously *via* a lateral tail vein. Intramuscular injections were made into the right medial thigh muscle. If lung colony counts were to be made, animals were sacrificed 6 weeks after injection and their lungs filled with Bouin's fixative *via* the trachea; the lungs were removed after 6 h fixation and stored in Bouin's solution. Colonies appeared as distinct pale nodules and all those >0·5 mm in diameter were scored; smaller colonies were judged to be macroscopically indistinct. The surface colonies were assumed to be representative of the total number of colonies throughout the lung.

In vitro *growth of tumour cells.*—$10^6$–$10^7$ tumour cells were grown at 37°C in 12 oz medical flats containing 30 ml of 80% Eagle's medium (+L-glutamine) and 20% calf serum. The proportion of cells in division 3 days after setting up the culture was estimated by incubating a suspension of the cells, obtained by trypsinization with 10 $\mu$g of colchicine for 6 h. After removal of the colchicine, the cells were fixed in 3 : 1 methanol : acetic acid and slides were prepared and scored for metaphase.

TABLE I.—*Colony Counts from Mice Injected with Mammary Tumour Cell Aggregates*

| No. of aggregates injected ($10^5$) | Mean no. of lung colonies |
|---|---|
| 0·3 | 7·0±1·0 |
| 0·5 | 10·4±1·4 |
| 1·0 | 18·9±2·6 |
| 1·5 | 31·6±4·6 |
| 2·0 | 39·1±5·1 |

### RESULTS

### *The colony forming efficiency (CFE) of tumour cell aggregates*

The routine separation of single cells from aggregates was shown to be unnecessary since the presence of excess single cells had no effect on the CFE of aggregates. The results presented in Table I and the Figure show the linear relation ship observed between the number of aggregates (containing up to 20 cells) injected into recipients and the number of macroscopic colonies that had developed in the lung within 6 weeks. The results are for one particular tumour but the linearity of the relationship has been confirmed in several other tumours.

Table II gives the CFE data for aggregates of different sizes for 2 tumours, A and B. It shows how the number of colonies per $10^5$ aggregates injected varies with the aggregate size profile and indicates a higher efficiency for the larger aggregates.

### *The CFE of single cell suspensions*

It was found repeatedly that single cells derived from spontaneous mammary tumours failed to produce colonies in the lungs of recipient mice. Thirteen different tumours were tested and up to $5 \times 10^6$ single cells were injected intravenously and no colonies were detected in over 130 mice, in contrast to the colonies that developed in recipients receiving an equal number of tumour cells injected as aggregates. It was therefore

FIG.—The relationship between inoculum size and lung colony formation for mammary tumour cell aggregates.

TABLE II.—*A Comparison of the CFE of Different Sized Aggregates*

| Tumour | Aggregate size profile | Total no. of cells injected ($10^5$) | Mean no. of lung colonies |
|---|---|---|---|
| A | 100%  | ($10^5$ aggregates injected) 8·7 | ≃300 |
| A | 100%  | 5·6 | 97·1±19·4 |
| B | 100%  | 9·9 | 6·0± 2·0 |
| B | 100%  a b c | 5·2 | 0·13±0·13 |

a = 2–4 cells
b = 5–15 cells
c = 16–20 cells of interest to determine how much the enzymatic dissociation method might have damaged single cells and 3 tests were performed:

First, the nigrosin dye exclusion method indicated that the viability of single cells was 70% some 2–3 h after dissociation. Second, short-term *in vitro* cultures of single cells showed that most of the cells had attached to the glass in 24 h, and after 2–3 days colchicine was added and showed that approximately 3·8% of the cells were dividing in 24 h. Third, $5 \times 10^5$ single tumour cells were injected either intramuscularly or intravenously and the former route produced palpable tumours in the thigh within 9 weeks, whereas no lung colonies developed following intravenous injections. It seems that the dissociation does little damage to the single cells, which remain viable and capable of proliferation, and so some other mechanism must be found to account for their apparently zero clonogenic potential compared with cells in close association as in aggregates.

It is well known that single cells derived from transplantable tumours are often clonogenic and it was decided to investigate, by serial passage, how rapidly single cells from spontaneous tumours became clonogenic. Two cell lines of spontaneous mammary tumours were initiated, one by passage in the thigh muscle and the other by passaging lung colony cells that had developed from intravenous injection of aggregates. At each passage dissociated single cells were assayed for their clonogenic ability and the results in Table III indicate how very few serial passages are required before single cells have a measureable CFE in the lung.

TABLE III.—*Effect of Transplantation on the Growth of Single Cells*

| Transplantation | Recipient mice with lung colonies |
|---|---|
| Intramuscular passage | |
| 1st–3rd | 0/6 |
| 4th | 2/6 |
| 5th | 6/6 |
| Intravenous passage | |
| 1st–2nd | 0/6 |
| 3rd | 4/6 |
| 4th | 6/6 |

Cells were transplanted *via* either an intravenous or an intramuscular route. Single cell suspensions of resultant tumours were prepared by enzymatic dissociation and recipient mice were injected intravenously with $5 \times 10^5$ cells.

### DISCUSSION

The main finding of the paper is that spontaneous tumour cell aggregates give rise to tumour colonies in the lungs but single cells do not. The single cells do, however, show *in vitro* proliferation and are capable of forming tumours *in vivo* when injected intramuscularly into the thigh. It is of interest that several authors (Southam, 1968; Wexler, Chretien and Ketcham, 1971) have reported finding differences in CFE for tumour cells in different *in vivo* sites. It is difficult to understand why the CFE of single cells is negligible compared with that of aggregates, although the efficiency of aggregates is itself very low when compared with the $TD_{50}$ values for cells from transplanted tumours. Neither immune nor genetic factors are likely to influence the potential colony growth since the tumour cells are comparatively non-immunogenic and are syngeneic with the host. There may be some co-operative effect between cells of an aggregate which enhances its CFE, but this seems unlikely since single cells can have their clonogenic potential markedly enhanced by 3–4 *in vivo* cell passages (Table III).

The size of cell aggregates is such that most will be trapped very efficiently in the lung capillaries. Despite this, the CFE of aggregates is still very low, which suggests that the growth of single cells is not likely to be unduly influenced by their failure to be trapped or to attach to the endothelium and form micro-thromboemboli.

There has been so little study of the clonogenicity of spontaneous tumours that generalization about possible factors affecting CFE is not possible although both this study and that of Watanabe (1954) for a spontaneous Dba mammary tumour show that such tumours contain a much lower proportion of clonogenic cells than do transplanted tumours.

The present work has shown that quantitative information can be gained about *in vivo* growth of spontaneous tumour cells which may prove useful in the future in highlighting possible important differences in clonogenic efficiency between spontaneous and transplanted tumours.

I am grateful to Professor Patricia J. Lindop for her constant guidance and to Drs J. E. Coggle and Jennifer Shewell and Mrs Krystyna Danielak for many helpful suggestions, and to Mr W. Hall for help with drawings.

The work was undertaken during the tenure of a Science Research Council grant.

### REFERENCES

BROWN, J. M. (1973) The Effect of Lung Irradiation on the Incidence of Pulmonary Metastases in Mice. *Br. J. Radiol.*, **46**, 613.

HILL, R. P. & BUSH, R. S. (1969) A Lung Colony Assay to Determine the Radiosensitivity of the Cells of a Solid Tumour. *Int. J. Radiat. Biol.*, **15**, 435.

SHAEFFER, J., EL-MAHDI, A. M. & CONSTABLE, W. C. (1973) Lung Colony Assay of Murine Mammary Tumor Cells Irradiated *in vivo* and *in vitro*. *Radiology*, **109**, 703.

SOUTHAM, C. M. (1968) Factors Influencing the Growth of Tumor Autotransplants. In *The Proliferation and Spread of Neoplastic Cells*. Baltimore: Williams & Wilkins Co. p. 581.

WATANABE, S. (1954) The Metastasizability of Tumor Cells. *Cancer, N.Y.*, **7**, 215.

WEXLER, H., CHRETIEN, P. B. & KETCHAM, A. S. (1971) The Fate of Circulating Methylcholanthrene Tumor Cells in Mice with Tumor Specific Immunity. *Cancer, N.Y.*, **28**, 641.

WITHERS, H. R. & MILAS, L. (1973) Influence of Pre-irradiation of Lung on Development of Artificial Pulmonary Metastases of Fibrosarcoma in Mice. *Cancer Res.*, **33**, 1931.